# Two new crane fly species of the subgenus Vestiplex Bezzi, 1924 (Diptera, Tipulidae, *Tipula*) from Yunnan and Sichuan, China, with a key to species in the *immota* species group

**DOI:** 10.3897/zookeys.1040.64376

**Published:** 2021-05-26

**Authors:** Qiu-Lei Men, Pavel Starkevich, Aidas Saldaitis

**Affiliations:** 1 School of Life Sciences, Province Key Laboratory of the Biodiversity Study and Ecology Conservation in Southwest Anhui Province, Anqing Normal University, Anqing, Anhui 246011, China Anqing Normal University Anqing China; 2 Nature Research Centre, Akademijos 2, LT-08412 Vilnius, Lithuania Nature Research Centre Vilnius Lithuania

**Keywords:** Hypopygium, ovipositor, taxonomy, Tipulinae

## Abstract

Two new crane fly species, Tipula (Vestiplex) gongdangensis**sp. nov.** and T. (V.) dechangensis**sp. nov.** are described and illustrated based on materials collected in the Yunnan and Sichuan provinces, China. A key is provided to distinguish males of the new species from those of other species in the T. (V.) immota Alexander, 1935 species group which is proposed here for the first time.

## Introduction

The World fauna of the subgenus Vestiplex Bezzi, 1924 currently amounts to 176 described species, distributed throughout the Holarctic and Oriental regions (Oosterbroek 2021). The Chinese fauna of *Vestiplex* is richly represented, with 69 species and one subspecies (Oosterbroek 2021).

The subgenus Vestiplex can be recognized by females having a powerfully constructed and heavily sclerotized cercus, usually with a serrated ventral margin, although margins may be smooth in some of the Asiatic species (Alexander 1935, 1965; Alexander and Byers 1981). The hypogynial valve is short to rudimentary, in the shape of blades or plates, or filamentous (Starkevich et al. 2019a, 2020).

Some species of the subgenus Vestiplex have tergite 9 of the male hypopygium forming a shallowly concave and sclerotized saucer (Alexander 1935; Alexander and Byers 1981; Starkevich et al. 2020), while other species have tergite 9 divided by a pale membrane at the midline, with the posterior margin of the dorsal portion simple or bearing additional lobes, ventrally membranous or terminating in a pair of plates, sclerotized processes, armatures or flattened lobes (Alexander 1935; Alexander and Byers 1981; Men et al. 2017; Pilipenko et al. 2019; Starkevich et al. 2019a, b).

Two new species were detected while sorting and identifying specimens of *Vestiplex* from the Sichuan and Yunnan provinces, China. In the present paper, the new species are described and illustrated. The Tipula (V.) immota Alexander, 1935 species group is proposed here for the first time. A key to separate all known species in this species group is given.

## Materials and methods

Adult crane flies were collected at ultraviolet light and with an insect net, and preserved in 96% ethanol. Specimens were studied with a Nikon SMZ800 (Nikon, Japan) and an Olympus SZ61 (Olympus, Japan) stereomicroscopes. Images were taken with a Canon EOS 80D (Canon, Japan) mounted on an Olympus SZX10 (Olympus, Japan) stereomicroscope and with a KUY NICE (KUY, China) mounted on an Olympus SZ61 stereomicroscope. The genitalia were studied after boiling in a 10% NaOH solution for 5–10 minutes.

Descriptive terminology follows that of Ribeiro (2006) and Cumming and Wood (2017). The term “appendage of sternite 9” (A9S) is adopted from Mannheims (1963), and the terms “ventral lobe” and “dorsal lobe” of A9S were adopted from Gelhaus (2005).

Abbreviations for institutional collections used herein: **AQNU** = Anqing Normal University, Anqing, China; **NRC** = Nature Research Centre, Vilnius, Lithuania; **USNM** = United States National Museum of Natural History, Washington, D.C., USA; other abbreviation: **PS** = slide by Pavel Starkevich.

Abbreviations for terms of the terminalia: **adm**, adminiculum; **AIA**, anterior immovable apodeme; **ap**, anal plate; **A9S**, appendage of sternite 9, **bu**, bursa; **c**, cercus; **CG**, clasper of gonostylus; **dl**, dorsal lobe of appendage of sternite 9; **dp**, dorsal portion of tergite 9; **EA**, ejaculatory apodeme; **gcx**, gonocoxite; **h**, hypogynial valve; **LG**, lobe of gonostylus; **PIA**, posterior immovable apodeme; **s**, sternite; **sp**, spermatheca; **sp d**, spermathecal duct; **t**, tergite; **vl**, ventral lobe of appendage of sternite 9; **vp**, ventral portion of tergite 9.

## Taxonomy

### Tipula (Vestiplex) immota Alexander, 1935 species group

The *immota* group can be distinguished from other T. (Vestiplex) species by tergite 9 having 1) a pair of brown, inconspicuously protruded rounded lobes dorsally, located on either side of the midline, 2) posterior margin terminating into a pair of short, heavily blackened processes and 3) membranous, brown ventral portion with additional microscopically darkened dots.

Males of the *immota* group can also be recognized by the following features: gonocoxite dorsally produced into a horn or spine; clasper of gonostylus terminating into an extended upper beak, dorsal crest nearly rectangular or angular, with a blackened ridge originating from the dorsal corner and a suffused black rim along dorsal surface; lower beak absent; sternite 9 with dorsal lobe of A9S short, fused basally with ventral lobe; adminiculum flattened basally and dilated, with median portion distinctly protruded. The female is known only for T. (V.) dechangensissp. nov. and is characterized by a smooth cercus and a short, blackened, plate-like hypogynial valve.

The *immota* group is close to the *bicornigera* species group (Starkevich et al 2019a). Both groups are characterized by an armed gonocoxite, tergite 9 with additional extensions on posterior margin, and dorsal lobe of A9S reduced. The *bicornigera* group can also be separated by the hypertrophic ventral portion of tergite 9, the absence of protruded lobes on the dorsal surface, and posteromedian lobes having the same level of sclerotizasion as the rest of the tergal surface, not heavily blackened as in the *immota* species group.

#### Tipula (Vestiplex) dechangensis

Taxon classificationAnimaliaDipteraTipulidae

sp. nov.

651B4F6C-1BB3-55BE-BCCF-A703FC625BB8

http://zoobank.org/59772C00-8AC4-4499-A956-AF7B340E2010

Figs 1–2 , 3–9 , 10–15 , 16–20 , 21 , 36

##### Type material.

***Holotype*:** male, **China**, Sichuan, road Dechang/Miyi, 27°05.34'N, 102°01.40'E, alt. 2100 m, 10 May 2018, R. Butvila & A. Saldaitis (NRC); preserved in ethanol.

***Paratypes*:** 3 males, 2 females, male genitalia slide No. PS0421m, wing slide No. PS0440m, female genitalia slide No. PS0422f, same data as holotype (NRC); preserved in ethanol.

##### Comparative material examined.

Tipula (V.) bicornuta Alexander, 1920: Holotype, male, **China**, Taiwan, Funkiko, 21 April 1917, pinned, T. Shiraki (USNM); antenna, wing and genitalia on slide (USNM). Tipula (V.) immota Alexander, 1935: Holotype, male, **China**, Sichuan, Kwanhsien, alt. 2000–4000 ft, 15–31 May 1933, Graham (USNM); pinned; paratype, male, topotypic (USNM), antenna, leg, wing and genitalia on slide (USNM).

##### Diagnosis.

Tipula (V.) dechangensissp. nov. can be recognized by the following combination of characters: body yellow, abdomen with basal segments yellow, tergites trivittate, distal segments, including hypopygium, dark brown; male antenna reaching base of abdomen if bent backward. Hypopygium with gonocoxite apically with a black spine that is curved at tip; tergite 9 divided at midline by a pale membrane, posterior margin with U-shaped notch, dorsal surface with pair of brown, inconspicuously protruded, rounded lobes, posterior margin terminating with a pair of black and short, wedge-shaped processes; adminiculum with a rounded preapical incision in lateral view. Female with cercus nearly straight, outer margin smooth, without visible serration, hypogynial valve in the shape of short brown plates, obtuse at apex.

##### Description

(Figs 1–20). Adult male (Fig. 1) (n = 4). Lengths: body 11.3–13.2 mm, wing 14.3–15.5 mm, antenna 5.1–6.6 mm.

**Figures 1, 2.**
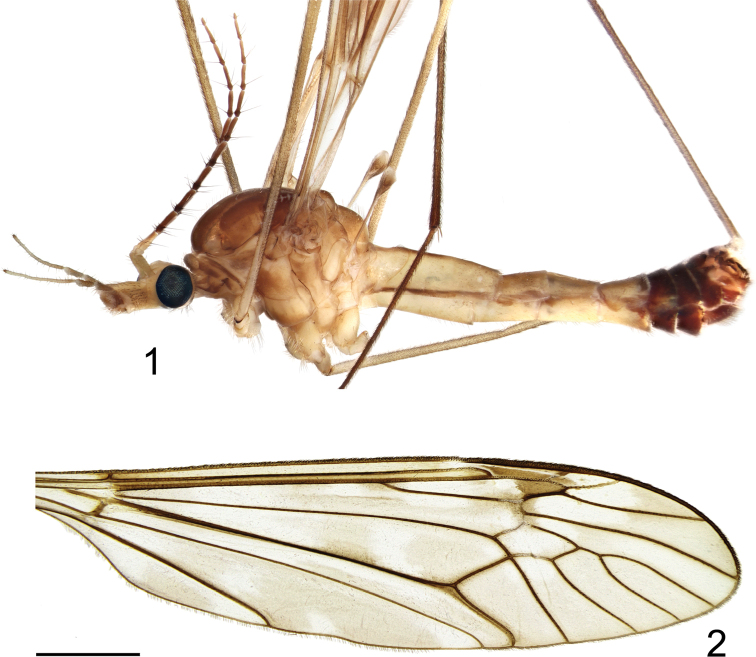
Tipula (Vestiplex) dechangensissp. nov. **1** holotype male, lateral view **2** wing. Scale bar: 2.0 mm.

***Head*.** Yellowish, vertex and occiput yellowish with dark brown median line. Rostrum yellowish, with short nasus. Antenna 13-segmented, elongate, if bent backward reaching base of abdomen. Scape, pedicel and first flagellomere yellow; following flagellomeres basally brown, apically light brown. Each flagellomere except first slightly enlarged at base (Fig. 1). Apical flagellomere small, reduced. Verticils shorter than their corresponding segments. Palpus brownish-yellow.

***Thorax*.** Pronotum light brown, with darker median line. Prescutum and presutural scutum brownish, with four brown longitudinal stripes. Interspaces grey, median pair fused anteriorly, with anterior margins inconspicuously bordered by darker brown. Scutum grey, scutal lobes each with two brown spots. Scutellum and mediotergite grey, with dark brown median line. Pleura yellowish, thinly grey-dusted (Fig. 1). Leg with coxa and trochanter yellow; femur yellow, with darkened tip; tibia and tarsal segments dark brown; tarsal claw with tooth (Fig. 1). Wing light brown (Fig. 2), cell sc not darker than ground colour, stigma dark brown, variegated by light areas including apical area of cell c and medial area of first cell of cell r_1_, a light band across apical area of cell r_1_, base of cells r_3_, r_4_ and r_5_, and median area of discal cell; remaining light area including base and apical areas of bm. Wing venation: R_1_ complete, R_2+3+4_ subequal in length to R_3_, R_4_ as long as Rs, R_5_ curved in apical half, r-m as long as base of R_5_, discal cell narrow, at least 3 times as long as petiole of cell m_1_, cell m_1_ more than 4× longer than its petiole. Halter pale yellow, knob brown basally, pale yellow apically.

***Abdomen*.** Abdominal segments 1–6 yellow, tergites trivittate, rest of segments, including hypopygium, brown (Fig. 1).

**Figures 3–9.**
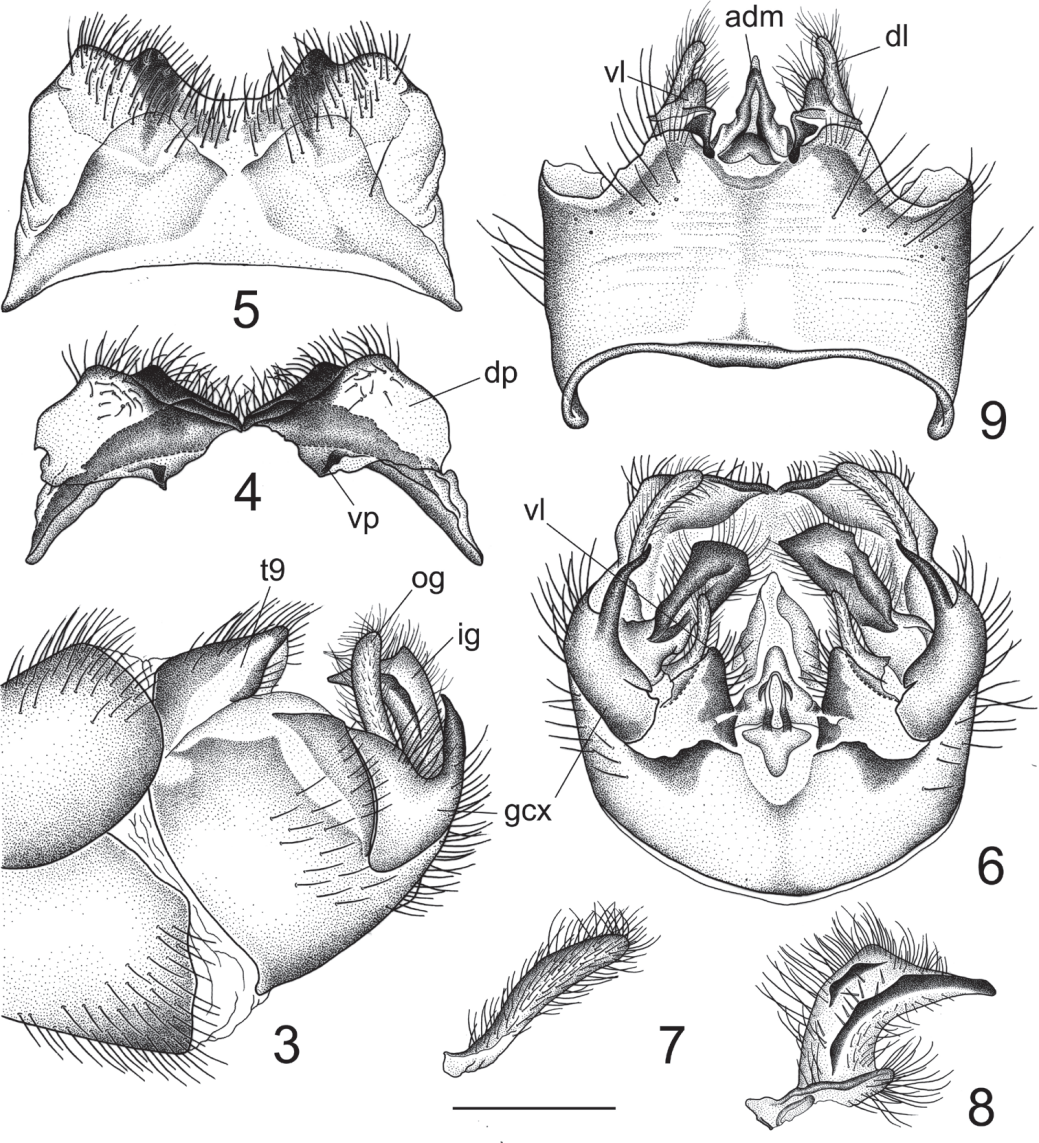
Male terminalia of Tipula (Vestiplex) dechangensissp. nov., holotype **3** hypopygium, lateral view **4** tergite 9, caudal view **5** tergite 9, dorsal view **6** hypopygium, caudal view **7** lobe of gonostylus (left) **8** clasper of gonostylus (left), lateral view **9** hypopygium, caudal view. Scale bars: 0.5 mm (**10–13**); 1.0 mm (**14, 15**).

**Figures 10–15.**
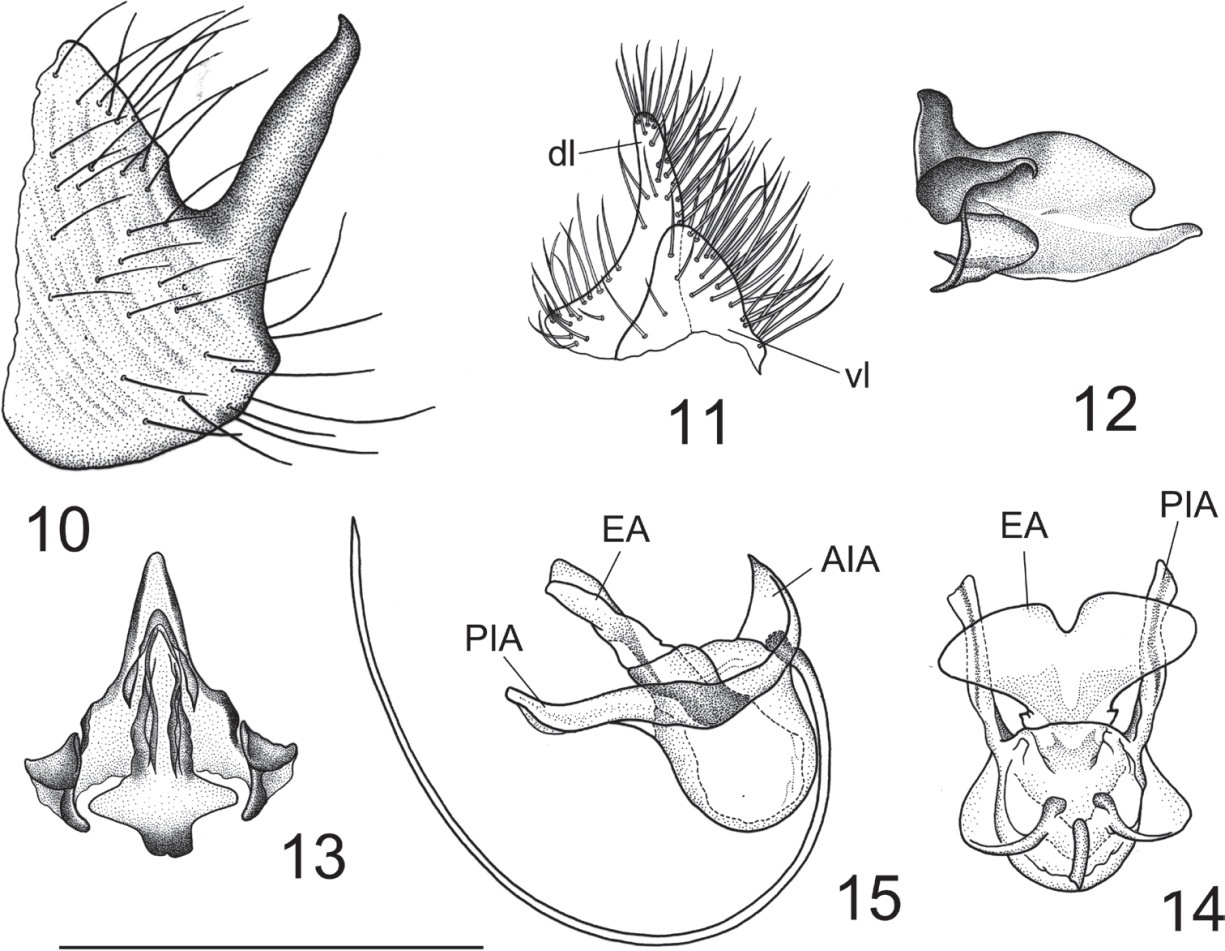
Male terminalia of Tipula (Vestiplex) dechangensissp. nov., holotype **10** right gonocoxite, lateral view **11** lobes of appendage of sternite 9 **12** adminiculum, lateral view **13** adminiculum, ventral view **14** sperm pump, dorsal view **15** sperm pump, lateral view.

***Hypopygium*** (Figs 3–15). Tergite 9 and sternite 9 totally separated. Tergite 9 completely divided at midline by a pale membrane (Fig. 5). Dorsal portion of tergite 9 laterally pale yellow, medially with a pair of brown, inconspicuously protruded, rounded lobes located on either side of midline; posterior margin covered with setae, medially with a U-shaped notch and a pair of black and short, wedge-shaped processes; posterolateral margin rounded (Figs 4, 5). Ventral portion membranous, brown, each half triangular at margin (Fig. 5). Gonocoxite not fused with sternite 9, posterior part produced into a dorsally-directed spine (Figs 3, 6, 10). Lobe of gonostylus narrowed, slightly curved, finger-shaped (Fig. 7). Clasper of gonostylus yellow, terminating in an extended upper beak; dorsal crest nearly rectangular with short, blackened ridge generated from the dorsal corner; a suffused black rim along dorsal surface; lower beak absent; base with short lobe and covered with setae (Figs 3, 6, 8). Sternite 9 with ventral lobe of A9S dark brown, nearly triangular, covered with setae (Fig. 11). Dorsal lobe of A9S yellow, densely covered with setae, short, finger-shaped, fused with ventral lobe at base (Fig. 11). Adminiculum triangular in ventral view, basally flattened and dilated (Fig. 12); median portion, before apex, distinctly protruded with margin raised at base; with a rounded preapical incision in lateral view, apex narrowed (Fig. 13). Sperm pump with ejaculatory apodeme fan-shaped, with a small V-shaped notch medially (Fig. 14). Posterior immovable apodeme of same length as ejaculatory apodeme, with paired arms curved dorsally, terminating in an acute apex in lateral view (Fig. 15). Anterior immovable apodeme broad, flattened, obtuse in dorsal view. Aedeagus more than 3.5 times as long as sperm pump (Fig. 15).

**Female.** Adult (n = 2). Lengths: body 18.4–20.1 mm, wing 15.1–16.8 mm, antenna 2.9–3.1 mm.

Generally similar to male. Antenna yellow, if bent backward reaching presutural scutum. Flagellar segments, except first, slightly enlarged at base. Abdomen yellow, tergites trivittate, with distinct dorsal stripe.

**Figures 16–20.**
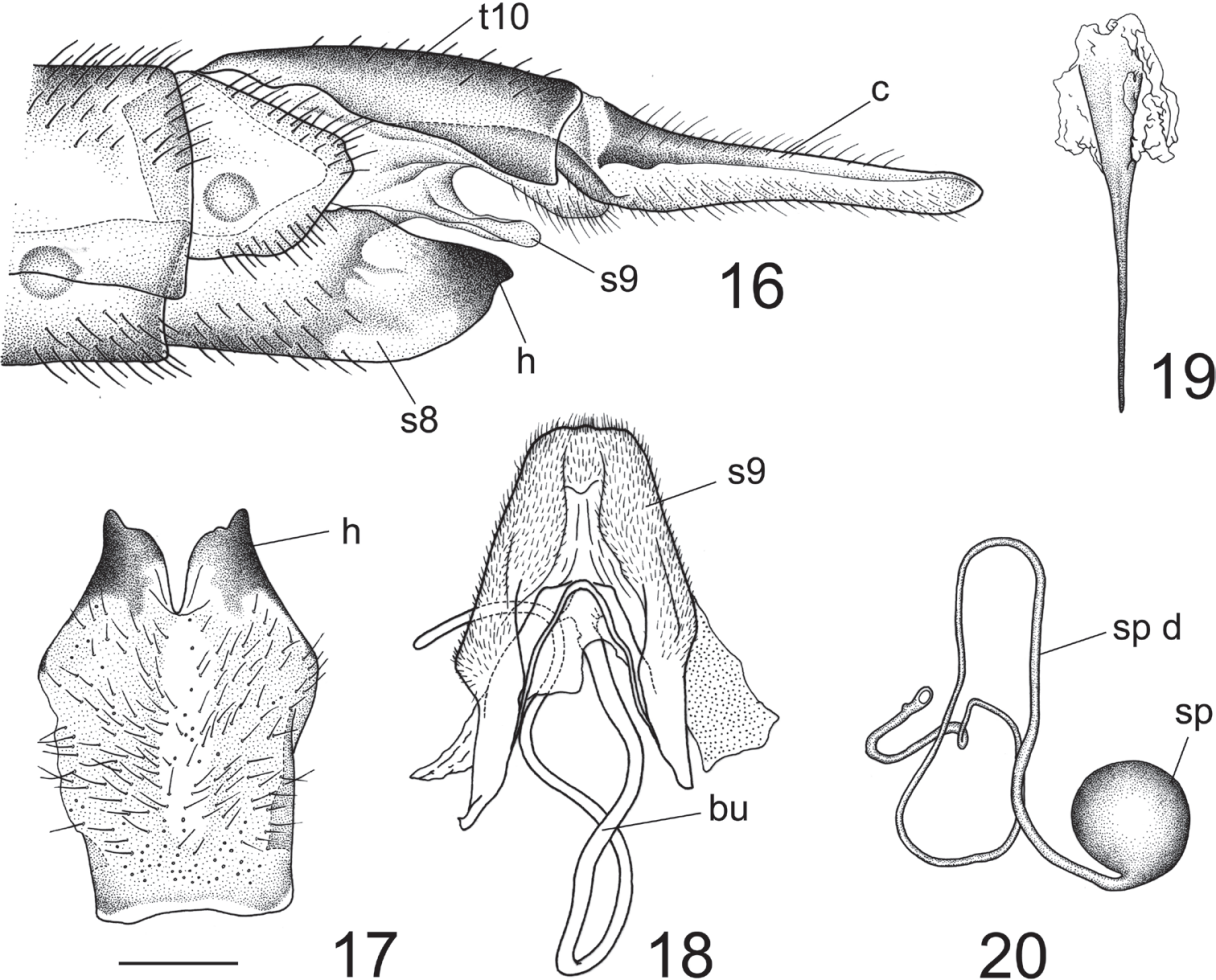
Female terminalia of Tipula (Vestiplex) dechangensissp. nov. (paratype) **16** ovipositor, left lateral view **17** eighth sternite with hypogynial valves, ventral view **18** sternite 9 with bursa **19** furca **20** spermatheca with spermathecal duct. Scale bars: 1.0 mm (**16, 17**); 0.5 mm (**18–20**).

***Ovipositor*** (Figs 16–20). Tergite 10 shiny yellow. Cercus yellow, nearly straight, with tip obtuse and outer margin smooth (Fig. 16). Sternite 8 yellow, with hypogynial valve brown (Figs 16, 17).

Hypogynial valve in the shape of short plate, brown (Fig. 17). Posterior margin of hypogynial valve medially incised, terminating in an obtuse apex in ventral view. Posterior part of sternite 9 covered with short filaments, shovel-shaped, medially with a groove, posterior margin rounded (Fig. 18). Anterior part of sternite 9 narrow, nearly straight (Fig. 18). Furca long, posteriorly flattened, anteriorly narrow (Fig. 19). Three spermathecae, spherical (Fig. 20).

##### Biology and distribution.

**Figure 21.**
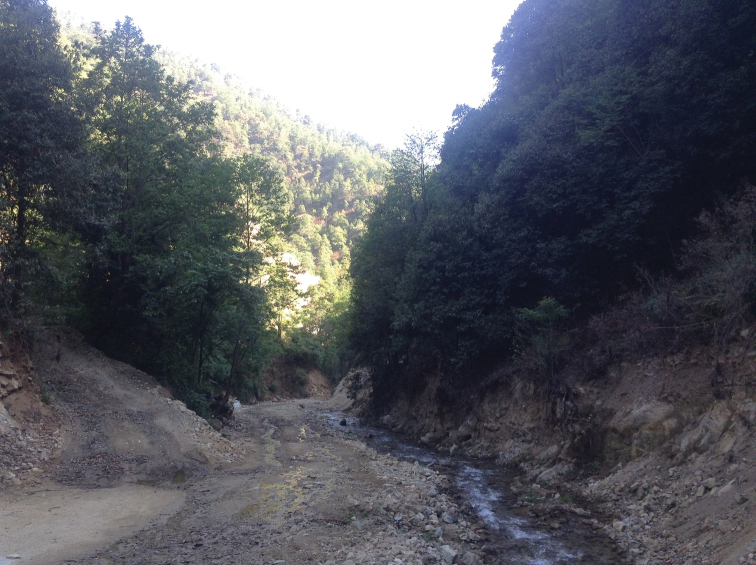
Type locality of Tipula (Vestiplex) dechangensissp. nov. China, Sichuan, road Dechang/Miyi, alt. 2100 m.

The new species is known from the Dechang, located at the eastern edge of the Tibetan plateau in Sichuan Province, China (Fig. 36). Two males and three females were collected at ultraviolet light at the beginning of May, at an altitude of around 2100 m. The new species was collected in the dry valley of a small river, with slopes covered by mixed forest dominated by various broad-leaved trees such as oaks (*Quercus dentata* Thunberg, *Q. glauca* Thunberg), poplars (*Populus cathayana* Rehder, *P. simonii* Carrière), elm (*Ulmus parvifolia* Jacquin), rhododendrons (*Rhododendron brachycarpum* G. Don, *R. dauricum* Linnaeus), and bamboos (*Phyllostachys* ssp., *Borinda* ssp., *Fargesia* spp.) (Fig. 21).

##### Etymology.

The new species name is derived from the type locality, Dechang, in Sichuan, China.

##### Disscussion.

Tipula (V.) dechangensissp. nov. is most similar to T. (V.) gongdangensissp. nov. in body and antenna colouration, and in the shape of the gonocoxite, the clasper of the gonostylus and the dorsal lobe of A9S. These two species can be separated by following details of tergite 9: posterior margin with U-shaped notch, black processes wedge-shaped, and posterolateral corner without extension in T. (V.) dechangensissp. nov.; posterior margin broadly emarginated, without U-shaped notch, black processes nearly triangular, and posterolateral corner extended in T. (V.) gongdangensissp. nov. Both species can be also separated by differences in the adminiculum, which has a preapical incision in T. (V.) dechangensissp. nov. that is absent in T. (V.) gongdangensissp. nov., and by the ventral lobe of A9S, which is well developed in T. (V.) dechangensissp. nov. and indistinct in T. (V.) gongdangensissp. nov.

#### Tipula (Vestiplex) gongdangensis

Taxon classificationAnimaliaDipteraTipulidae

sp. nov.

188D53B0-4DA7-5FD9-BC2A-7474C3055798

http://zoobank.org/D6CD9F93-8220-4241-8979-3C7453D7C492

Figs 22–23 , 24–34 , 35 , 36

##### Type material.

***Holotype*:** male, **China**, Yunnan, Gongshan County, Bingzhongluo, Gongdangshenshan, 27.97°N, 98.66°E, 12 June 2019, leg. Men QL & Lv L (AQNU), preserved in ethanol.

***Paratypes*:** 23 males, topotypic (AQNU), preserved in ethanol.

##### Diagnosis.

Tipula (V.) gongdangensissp. nov. can be recognized by the following combination of characters: body yellow, abdomen with basal segments yellow, tergites trivittate, distal segments, including hypopygium, dark brown; male antenna reaching base of abdomen if bent backward. Hypopygium with gonocoxite armed with a black spine; tergite 9 divided at midline by a pale membrane, broadly emarginated at posterior margin, dorsal surface with a pair of brown, inconspicuously protruded, rounded lobes, posterior margin terminating with a pair of short, black, triangular processes. Adminiculum with median portion before apex distinctly protruded, apex curved, acute in lateral view.

##### Description

(Figs 22–34). Adult male (Fig. 22) (n = 24). Lengths: body 10.5–11.2 mm, wing 14.0–14.2 mm, antenna 5.6–5.9 mm.

**Figure 22, 23.**
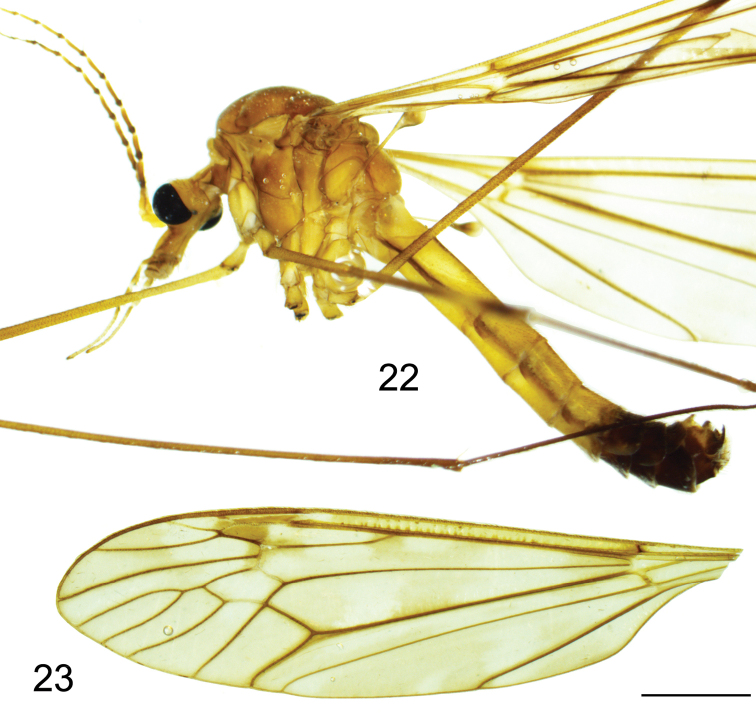
Tipula (Vestiplex) gongdangensissp. nov. **22** holotype male, lateral view of holotype **23** wing. Scale bar: 2.0 mm.

***Head*.** Yellowish, vertex and occiput yellowish, medially with narrow dark brown line. Rostrum yellowish, with short nasus densely covered with black setae. Antenna 13-segmented, elongate, if bent backward reaching base of abdomen. Scape, pedicel and first flagellomere yellow; following flagellomeres basally brown, apically light brown, producing an indistinct bicoloured appearance (Fig. 22). Each flagellomere, except first, slightly enlarged at base, segments progressively shortened and narrowed. Apical flagellomere small, reduced. Verticils shorter than their corresponding segments. Palpus brownish-yellow.

***Thorax*.** Pronotum light brown, with darker median area. Prescutum and presutural scutum brownish, with four brown longitudinal stripes. Interspaces between median and lateral stripes grey, with light and short setae. Median pair with anterior margins and inner margins on apical 1/4 suffused with black. Scutum grey, scutal lobes grey-pruinose, each with two brown spots. Scutellum and mediotergite grey-pruinose, with dark brown median line. Pleura yellowish, thinly dusted with brown (Fig. 22). Leg with coxa and trochanter yellow; femur yellow with tip dark brown; tibia and tarsal segments dark brown; tarsal claw with a tooth. Wing light brown (Fig. 23), cell sc darker than ground colour, stigma dark brown with a light area at base, variegated by light areas, including apical area of cell c and median area of 1^st^ cell of cell r_1_, a light band across apical area of cell r_1_, base of cells r_3_, r_4_ and r_5_, and median area of discal cell; remaining area light, including base and apical areas of cell bm, and median and apical areas of cells cua and cup. Wing venation: R_1_ atrophied in basal half, R_2+3+4_ slightly shorter than R_3_, R_4_ distinctly shorter than Rs, R_5_ curved in apical half, r-m distinctly longer than base of R_5_, discal cell narrow, 3× as long as petiole of cell m_1_, cell m_1_ more than 4× as long as its petiole. Halter with stem yellow, knob brown, with apical part lighter.

***Abdomen*.** Abdominal segments 1–6 yellow, with brown lateral and median stripes; remaining segments, including hypopygium, brown (Fig. 22).

**Figures 24–34.**
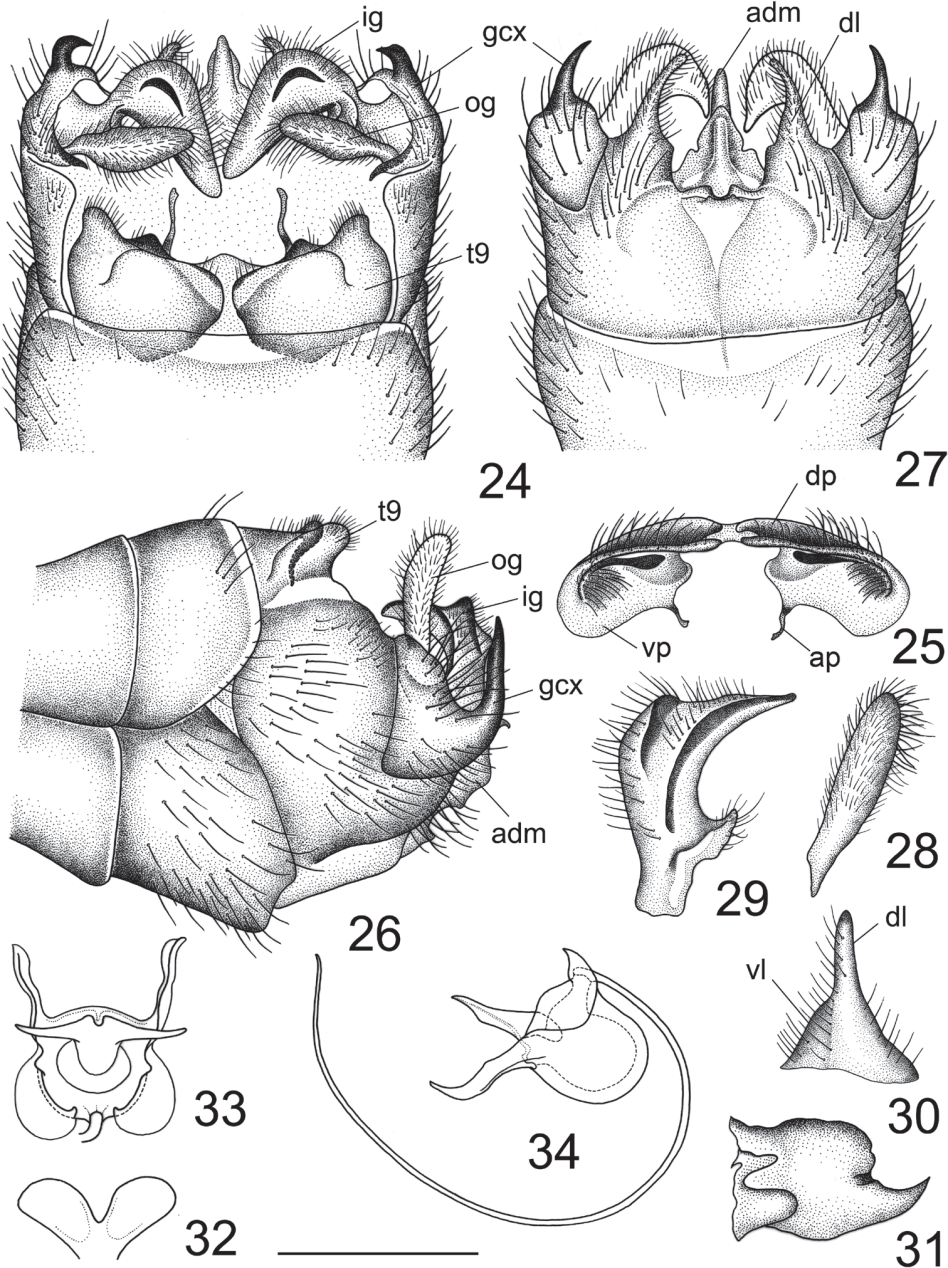
Male terminalia of Tipula (Vestiplex) gongdangensissp. nov., holotype **24** hypopygium, dorsal view **25** tergite 9, caudal view **26** hypopygium, lateral view **27** hypopygium, ventral view **28** lobe of gonostylus (left) **29** clasper of gonostylus (left), lateral view **30** lobes of appendage of sternite 9 **31** adminiculum, lateral view **32** ejaculatory apodeme **33** sperm pump, dorsal view **34** sperm pump, lateral view. Scale bars: 0.5 mm (**23–30**); 0.8 mm (**31–33**).

***Hypopygium*.** (Fig. 24–34). Tergite 9 and sternite 9 totally separated (Fig. 24). Tergite 9 completely divided at midline by a pale membrane (Fig. 24). Dorsal portion of tergite 9 medially with a pair of brown, inconspicuously protruded, rounded lobes, located on either side of midline; posterior margin emarginated, with a pair of black, short, nearly triangular processes, posterolateral margin extended into rounded lobes that are covered with setae. Ventral portion membranous, brown, with darker areas provided with brown microscopic dots; anal plates narrowed, attached to rounded margins of ventral portion (Fig. 25). Gonocoxite entirely cut off from sternite 9, basally covered with setae, dorsally produced into a slightly curved spine with an acute tip (Figs 26, 27). Lobe of gonostylus slightly flattened in middle (Fig. 28). Clasper of gonostylus yellow, upper beak extended, dorsal crest angular, with a black ridge originating from the dorsal corner; with a suffused black rim along dorsal surface; lower beak absent; base with triangular lobe and covered with setae (Figs 24, 26, 29). Sternite 9 with ventral lobe of A9S dark brown, nearly triangular, covered with setae (Figs 27, 30). Dorsal lobe of A9S yellow, densely covered with setae, short, narrowed towards apex, fused with ventral lobe at base (Fig. 30). Adminiculum triangular in ventral view, basally flattened and dilated (Fig. 27); median portion, before apex, distinctly protruded; apex curved, acute at tip (Fig. 31). Sperm pump (Figs 32–34) with ejaculatory apodeme V-shaped, each arm expanded and rounded apically (Fig. 32). Posterior immovable apodeme with strongly curved paired arms, terminating in an acute apex in lateral view (Fig. 34). Anterior immovable apodeme broad, flattened, round in dorsal view (Fig. 33). Aedeagus more than 4× as long as sperm pump (Fig. 34).

**Female.** Unknown.

##### Biology and distribution.

**Figure 35.**
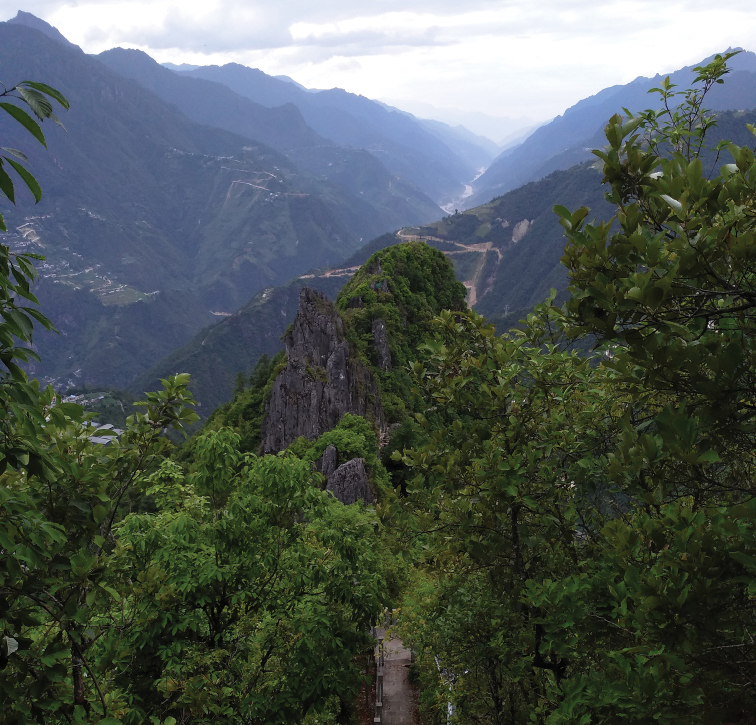
Type locality of Tipula (Vestiplex) gongdangensissp. nov. China, Yunnan, Gongshan County, Bingzhongluo, Gongdangshenshan.

**Figure 36.**
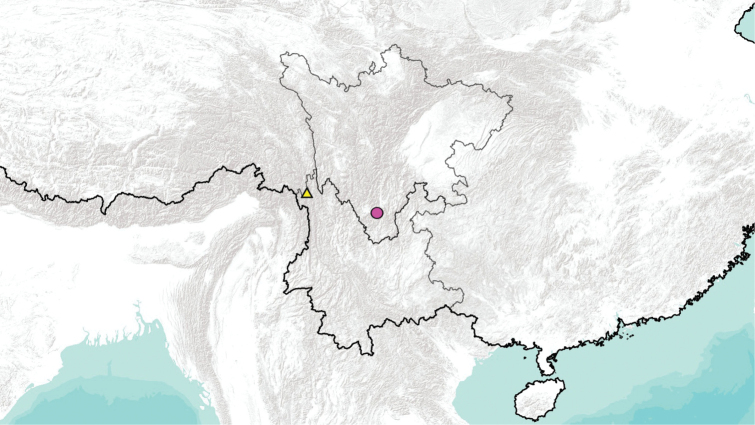
Collecting sites of Tipula (Vestiplex) in China: Sichuan, Tipula (V.) dechangensissp. nov. (triangle); Yunnan, Tipula (V.) gongdangensissp. nov. (circle).

A total of 24 males were collected with insect nets around the middle of June, 2019 on Gongdang Mountain, located in the south of Bingzhongluo town of Nujiang Lisu Autonomous Prefecture, Yunnan Province (Fig. 36). The new species was collected at altitudes of approximately 2000–2400 m, in mixed mountain forest dominated by various deciduous trees and bushes (Fig. 35).

##### Etymology.

The specific epithet refers to the type locality, Gongdang Mountain, Yunnan, China.

##### Discussion.

Tipula (V.) gongdangensissp. nov. and T. (V.) dechangensissp. nov. are closely related to T. (V.) dashahensis (Yang et al. 2005: p. 381, fig. 1A–C) based on the shape of the clasper of the gonostylus and of the dorsal lobe of A9S, but they differ by the shape of gonocoxite, which is stout, horn-shaped in T. (V.) dashahensis and is slender, spine-shaped in T. (V.) dechangensissp. nov. and T. (V.) gongdangensissp. nov. The two new species can also be separated by the intermediate stripes on the prescutum and presutural scutum, which are fused in T. (V.) dashahensis and separated, except at the anterior margin, in both of the newly described species.

**Figures 37, 38.**
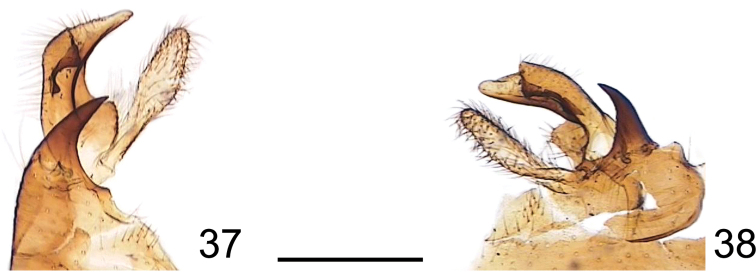
Genitalia slides of C. P. Alexander (USNM) **37**Tipula (Vestiplex) bicornuta, holotype, gonocoxite, lobe of gonostylus and clasper of gonostylus **38**Tipula (V.) immota, paratype, gonocoxite, lobe of gonostylus and clasper of gonostylus. Scale bar: 0.05 mm (**37, 38**).

#### Key to species (male) of the *immota* species group

**Table:** 

1	Gonocoxite apically stout, horn-shaped, broad at base, gradually narrowing toward apex (Figs 37, 38; Yang et al. 2005: p. 381, fig. 1A)	**2**
–	Gonocoxite apically with a slender spine (Figs 3, 26)	**4**
2	Prescutum and presutural scutum with fused intermediate stripes (Yang et al. 2005)	**T. (V.) dashahensis Yang, Zhu & Liu, 2005**
–	Prescutum and presutural scutum with intermediate stripes separated, confluent only at anterior margin	**3**
3	Flagellum bicolorous. Femora brownish-yellow, blackened at tip, preceded by a slightly brighter ring. Clasper of gonostylus slightly curved, beak broadened, rounded at tip; dorsal margin extended into indistinct teeth (Fig. 37)	**T. (V.) immota Alexander, 1935**
–	Flagellar segments black basally and dark brown distally. Femora brown, tip broadly dark brown. Clasper of gonostylus bent almost at a right angle, with beak narrowing toward apex; dorsal margin extended into two distinct teeth (Fig. 38)	**T. (V.) bicornuta Alexander, 1920**
4	Gonocoxite with curved, spine-shaped tip (Fig. 3). Tergite 9, at posterior margin, with median U-shaped notch; posterolateral margin of tergite 9 not extended (Fig. 5)	**T. (V.) dechangensissp. nov.**
–	Gonocoxite with tip not curved (Fig. 26). Tergite 9 broadly emarginated at posterior margin, without median U-shaped notch; posterolateral margin of tergite 9 extended into short lobes (Fig. 24)	**T. (V.) gongdangensissp. nov.**

## Supplementary Material

XML Treatment for Tipula (Vestiplex) dechangensis

XML Treatment for Tipula (Vestiplex) gongdangensis
